# A new species of blunt-headed vine snake (Colubridae, *Imantodes*) from the Chocó region of Ecuador

**DOI:** 10.3897/zookeys.244.3950

**Published:** 2012-11-27

**Authors:** Omar Torres-Carvajal, Mario H. Yánez-Muñoz, Diego Quirola, Eric N. Smith, Ana Almendáriz

**Affiliations:** 1 Escuela de Biología, Pontificia Universidad Católica del Ecuador, Avenida 12 de Octubre y Roca, Apartado 17–01–2184, Quito, Ecuador; 2 Museo Ecuatoriano de Ciencias Naturales, calle Rumipamba 341 y Avenida de los Shyris, Apartado 17–07–8976, Quito, Ecuador; 3 Department of Biology and Amphibian & Reptile Diversity Research Center, The University of Texas at Arlington, Arlington, TX 76019, USA; 4 Instituto de Ciencias Biológicas, Escuela Politécnica Nacional, Ladrón de Guevara E11–253, Quito, Ecuador

**Keywords:** Chocó, Dipsadinae, Ecuador, *Imantodes*, snakes, systematics

## Abstract

We describe a new species of *Imantodes* from the Chocó region of northwestern Ecuador. The new species differs most significantly from all other congeners in lacking a loreal scale. We analyze the phylogenetic relationships among species of *Imantodes* based on two mitochondrial genes, and postulate that the new species and *Imantodes lentiferus* are sister taxa. A key to the species of *Imantodes* from Ecuador is presented.

## Introduction

The New World colubrid snake clade Dipsadinae Bonaparte 1838 includes more than 400 extant species assigned to approximately 25 taxa traditionally ranked as genera (Daza et al. 2009; Zaher et al. 2009). Most members of the Dipsadinae have unilobed (or nearly unilobed), unicapitate hemipenes, with the *sulcus spermaticus* dividing distally (Zaher et al. 2009). One of the most remarkable dipsadine genera is *Imantodes*. Its long, thin body, disproportionately slender neck, and blunt head, makes easy to distinguish *Imantodes* from all other New World snakes. This genus includes six currently recognized species (*Imantodes cenchoa*, *Imantodes gemmistratus*, *Imantodes inornatus*, *Imantodes lentiferus*, *Imantodes phantasma*, and *Imantodes tenuissimus*) commonly known as blunt-headed vine snakes, occurring from Mexico to Argentina (Myers 1982).

Studies on phylogenetic relationships and species limits among dipsadines are scarce. However, recent work provides strong evidence from DNA sequence data for a clade containing *Imantodes* and *Leptodeira*, although monophyly of *Imantodes* remains controversial (Daza et al. 2009; Mulcahy 2007). Futhermore, *Imantodes gemmistratus* as currently circumscribed appears to be paraphyletic (Daza et al. 2009; Mulcahy 2007). Future studies with increased taxon and character sampling will probably clarify the phylogenetic relationships and species limits within *Imantodes*.

Three species of blunt-headed vine snakes are known from Ecuador; *Imantodes inornatus* and *Imantodes lentiferus* occur west and east of the Andes, respectively, whereas *Imantodes cenchoa* is known from both versants (Torres-Carvajal and Salazar-Valenzuela 2012). In this paper we describe a new species of *Imantodes* from northwestern Ecuador and infer its phylogenetic affinities to other species in the genus as currently circumscribed.

## Materials and methods

### Morphological data

All type specimens of the new species described in this paper are listed in the type series below, and were deposited at the Museo de Zoología, Pontificia Universidad Católica del Ecuador, Quito (QCAZ), the Museo Ecuatoriano de Ciencias Naturales, Quito (DHMECN), and the Amphibian & Reptile Diversity Research Center at The University of Texas at Arlington, USA (UTA). Specimens of other species of *Imantodes* examined in this study are listed in the appendix. Snout-vent length (SVL) and tail length (tL) measurements were recorded to the nearest millimeter. All other measurements were made with digital calipers and recorded to the nearest 0.01 mm. Sex was determined by noting the presence of hemipenes, everted or by tail dissection. Partially everted hemipenes were prepared following standard techniques (Pesantes 1994; Zaher 1999). Differences in scale counts between the new species and other species of *Imantodes* were evaluated with t-tests for normally distributed variables (i.e., Shapiro-Wilk test, P > 0.05), all of which had equal variances (i.e., F-test, P > 0.001). We used the program PAST 2.15 (Hammer et al. 2001) for all statistical tests.

### DNA Sequence Data

Total genomic DNA was digested and extracted from liver or muscle tissue using a guanidinium isothiocyanate extraction protocol. Tissue samples were first mixed with Proteinase K and lysis buffer and digested overnight prior to extraction. DNA samples were quantified using a Nanodrop® ND-1000 (NanoDrop Technologies, Inc), re-suspended and diluted to 25 ng/ul in ddH2O prior to amplification.

We amplified 1674 nucleotides (nt) encompassing two mitochondrial loci, NADH dehydrogenase subunit 4 (ND4, 651 nt) and cytochrome b (cyt-b, 1023 nt) from five individuals of *Imantodes cenchoa*, three of *Imantodes lentiferus*, three of the new species described herein and one of *Leptodeira septentrionalis*. Cyt-b was amplified using the primers Gludg, L14910, and H16064 (Burbrink et al. 2000; Parkinson et al. 2002), whereas ND4 was amplified using the primers ND4, LEU and ND412931L (Arévalo et al. 1994; Blair et al. 2009). Additionally, we used sequences of *Imantodes cenchoa*, *Imantodes gemmistratus*, *Imantodes inornatus*, *Imantodes lentiferus* and *Imantodes septentrionalis* from GenBank. Although monophyly of *Imantodes* has not been rigorously tested yet (see Discussion), for the purposes of this study we assume that *Imantodes* forms a clade and root our tree with *Leptodeira septentrionalis*. Gene regions of taxa included in phylogenetic analyses along with their GenBank accession numbers and locality data are shown in Table 1. Amplification of genomic DNA consisted of an initial cycle at 94 C for 3.5 min, 42 C for 1 min, and 68 C for 1.5 min, followed by 40 cycles of a denaturation at 94 C for 30 s, annealing at 52 C for 30 s, and extension at 72 C for 60 s, as well as a final extension at 72 C for 15 min.

**Table 1.** Vouchers, locality data, and GenBank accession numbers of taxa and gene regions included in this study. Asterisks indicate new sequences obtained for this study.

**Taxon**	**Voucher**	**Locality**	**Genbank accession number**	
			**Cyt-b**	**ND4**
*Imantodes cenchoa*	MPEGLJV 5763	Brasil: Para	EF078556	EF078508
*Imantodes cenchoa*	JMD 1616	Colombia: Chocó	GQ334486	GQ334587
*Imantodes cenchoa*	MHUA R-14290	Colombia: Antioquia	GQ334484	GQ334585
*Imantodes cenchoa*	MHUA R-14500	Colombia: Antioquia	GQ334485	GQ334586
*Imantodes cenchoa*	MVZ 149878	CostaRica: Limón	EF078553	EF078505
*Imantodes cenchoa*	QCAZ 11115	Ecuador: Santo Domingo de los Tsáchilas	*KC176244	*KC176256
*Imantodes cenchoa*	QCAZ 6300	Ecuador: Esmeraldas	*KC176248	*KC176260
*Imantodes cenchoa*	QCAZ 4207	Ecuador: Orellana	*KC176247	*KC176259
*Imantodes cenchoa*	UTA R-42360	Guatemala: Izabal	EF078554	EF078506
*Imantodes cenchoa*	SIUCR 03724	Panama: Cocle	EF078555	EF078507
*Imantodes cenchoa*	CORBIDI 3794	Peru: Tumbes	*KC176245	*KC176257
*Imantodes cenchoa*	CORBIDI 8823	Peru: San Martín	*KC176246	*KC176258
*Imantodes chocoensis * sp. n.	QCAZ 7978	Ecuador: Esmeraldas	*KC176249	*KC176261
*Imantodes chocoensis * sp. n.	QCAZ 7984	Ecuador: Esmeraldas	*KC176250	*KC176262
*Imantodes chocoensis * sp. n.	UTA R-60205	Ecuador: Esmeraldas	*KC176254	*KC176266
*Imantodes gemmistratus*	UTA R-45922	Guatemala: San Marcos	GQ334487	GQ334588
*Imantodes gemmistratus*	LSUMZ 39541	Mexico: Sonora	EF078558	EF078510
*Imantodes gemmistratus*	UTA R-51979	Mexico: Sinaloa	EF078557	EF078509
*Imantodes inornatus*	MHUA R-14540	Colombia: Antioquia	GQ334488	GQ334589
*Imantodes inornatus*	ASL 307	CostaRica	GQ334489	GQ334590
*Imantodes inornatus*	MVZ 204109	CostaRica: Cartago	EF078559	EF078511
*Imantodes inornatus*	MVZ 204110	CostaRica: Heredia	EF078560	EF078512
*Imantodes lentiferus*	MPEGLJV 5581	Brazil: Para	EF078562	EF078514
*Imantodes lentiferus*	MPEGLJV 6880	Brazil: Amazonas	EF078561	EF078513
*Imantodes lentiferus*	QCAZ 8377	Ecuador: Pastaza	*KC176251	*KC176263
*Imantodes lentiferus*	QCAZ 8488	Ecuador: Zamora Chinchipe	*KC176252	*KC176264
*Imantodes lentiferus*	QCAZ 9187	Ecuador: Morona Santiago	*KC176253	*KC176265
*Leptodeira septentrionalis*	MHUA R-14403	Colombia: Antioquia	GQ334528	GQ334632
*Imantodes septentrionalis*	QCAZ 10550	Ecuador: Esmeraldas	*KC176243	*KC176255

### Phylogenetic analyses

Editing, assembly, and alignment of sequences were performed with Geneious ProTM 5.3 (Drummond et al., 2010). Phylogenetic relationships were assessed under a Bayesian approach in MrBayes 3.2.0 (Ronquist and Huelsenbeck, 2003). The model of character evolution for each gene was obtained in JModeltest (Posada, 2008) under the Akaike information criterion. Genes were combined into a single dataset with two partitions, one per gene. Four independent analyses were performed to reduce the chance of converging on a local optimum. Each analysis consisted of five million generations and four Markov chains with default heating values. Trees were sampled every 1000 generations resulting in 5000 saved trees per analysis. Stationarity was confirmed by plotting the log-likelihood scores per generation in the program Tracer 1.2 (Rambaut and Drummond, 2003). Additionally, the standard deviation of the partition frequencies and the potential scale reduction factor (Gelman and Rubin, 1992) were used as convergence diagnostics for the posterior probabilities of bipartitions and branch lengths, respectively. Adequacy of mixing was assessed by examining the acceptance rates for the parameters in MrBayes and the effective sample sizes (ESS) in Tracer. After analyzing convergence and mixing, 500 trees were discarded as “burn-in” from each run. We then confirmed that the four analyses reached stationarity at a similar likelihood score and that the topologies were similar, and used the resultant 18,000 trees to calculate posterior probabilities (PP) for each bipartition on a 50% majority rule consensus tree.

## Results

The taxonomic conclusions of this study are based on the observation of morphological features and color patterns, as well as inferred phylogenetic relationships. We consider this information as species delimitation criteria following the general species concept of de Queiroz (1998, 2007).

### Imantodes chocoensis sp. n.

Proposed standard English name: Chocoan blunt-headed vine snakes

Proposed standard Spanish name: Cordoncillos del Chocó

urn:lsid:zoobank.org:act:D47B3B06-B8B2–4FDC-A54E-3A1F99091044

http://species-id.net/wiki/Imantodes_chocoensis

#### Holotype.

**Figure 1.**
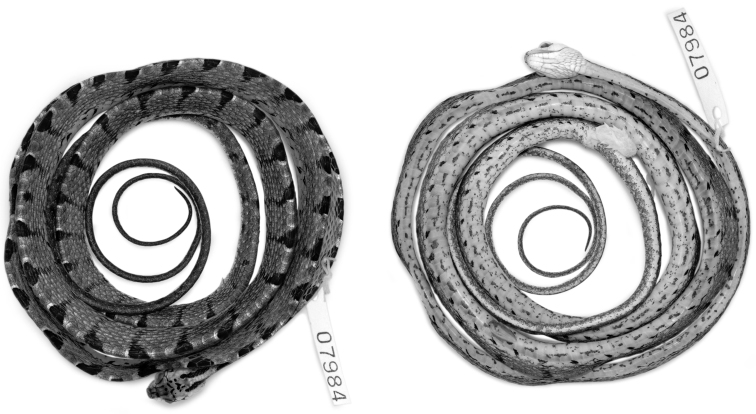
Holotype of *Imantodes chocoensis* sp. n. in dorsal (left) and ventral (right) views. Photographs by OTC.

**Figure 2:**
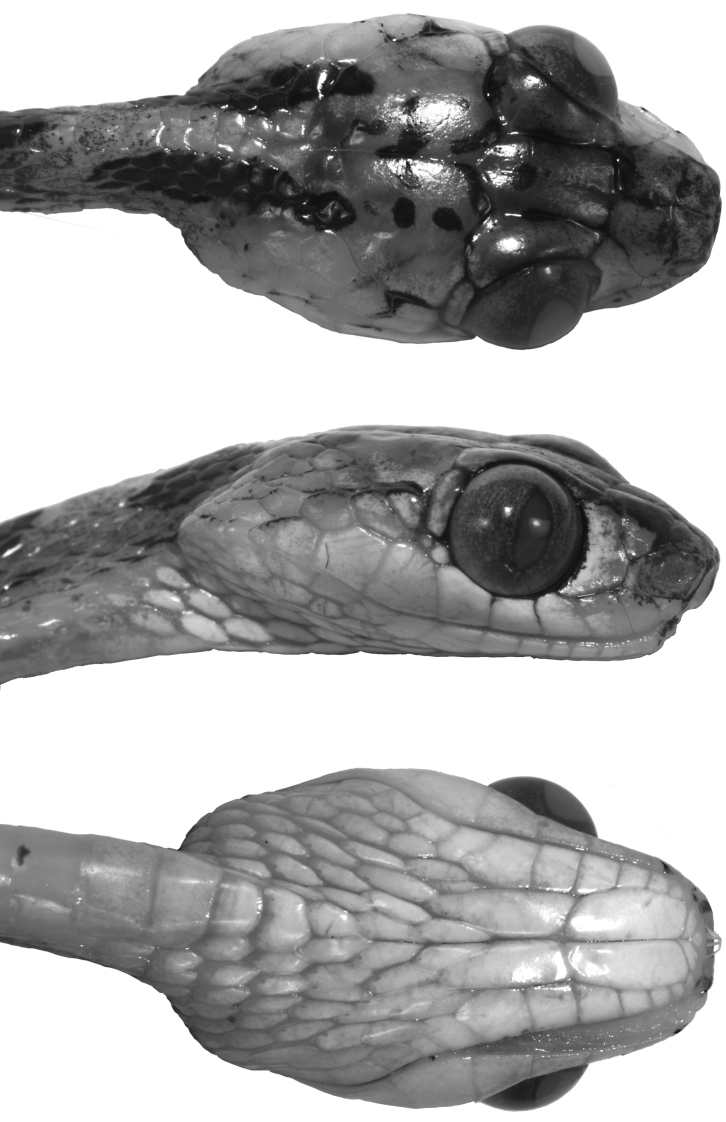
. Head of holotype of *Imantodes chocoensis* sp. n. in dorsal (top), lateral (middle) and ventral (bottom) views. Photographs by OTC.

– QCAZ 7984 (Figs. 1, 2), an adult male from 4 km N Durango, 1.0283°N, 78.5950°W (DD), 253 m, Provincia Esmeraldas, Ecuador, collected on 24 April 2007 by E. Carrillo-Ponce, I. G. Tapia, and E. E.Tapia.

#### Paratypes (6).

– ECUADOR: Provincia Carchi: DHMECN 6753, Río San Juan, 1.1858°N, 78.5006°W (DD), 243 m, collected on 12 September 2009 by M. Yánez-Muñoz, L. Oyagata, and M. Altamirano; DHMECN 6757, Sendero Awa, 1.1643°N, 78.5071°W (DD), 257 m, collected on 16 September 2009 by M. Yánez-Muñoz, L. Oyagata, and M. Altamirano. Provincia Esmeraldas: UTA R-60205, San Lorenzo-Santa Rita, 1.0321°N, 78.7138°W (DD), 115 m, collected on 21 March 2008 by M. Alcoser, R. Betancourt, P. Loaiza L., L. Oyagata, S. Ramírez J., J. W. Streicher, C. Tobar, and E. N. Smith; QCAZ 7978, same collection data as holotype; QCAZ 10185, 4 km W Alto Tambo, 0.91241°N, 78.5809°W (DD), collected on 18 December 2009 by S. Poe, L. Gray, and I. Latella; QCAZ 10710, Playa de Oro, Estero Pote and Estero Angostura, lower part of Cotacachi Cayapas Ecological Reserve, 0.8285°N, 78.7220°W (DD), collected on 27 November 1994 by E. Toral-Contreras, V. Ortiz, and F. Nogales.

#### Diagnosis.

**Figure 3.**
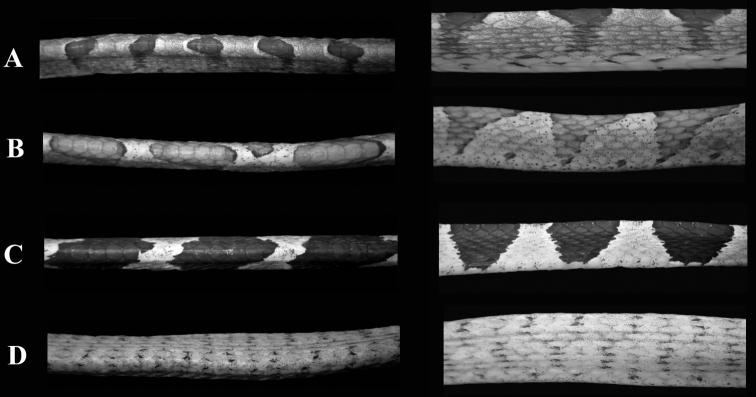
Body segments of species of *Imantodes* from Ecuador in dorsal (left) and lateral (right) views. **A** *Imantodes chocoensis* sp. n. (DHMECN 6753, paratype) **B** *Imantodes lentiferus* (DHMECN 8345) **C** *Imantodes cenchoa* (DHMECN 7826) **D** *Imantodes inornatus* (DHMECN 5661). Photographs by MYM.

*Imantodes chocoensis* differs from all other known congeners in lacking a loreal scale. It can be further distinguished from its sister species (see Phylogenetic relationships) *Imantodes lentiferus* by having 17 longitudinal rows of dorsal scales at midbody and at nearly one head length anterior to the cloaca (15 in *Imantodes lentiferus*), more ventrals (*t* = 7.27, *P* < 0.001), more subcaudals (*t* = -4.31, *P* < 0.001), more postoculars (2–3, mean = 2.43 ± 0.51; 1–2, mean = 1.81 ± 0.39 in *Imantodes lentiferus*), more infralabials (12–15, mean = 13.21 ± 0.80; 9–12, mean = 10.68 ± 0.60 in *Imantodes lentiferus*), and smaller dark blotches on dorsum (Fig. 3). Among other species of *Imantodes* known from Ecuador, the new species differs further from *Imantodes inornatus* (*N* = 2–3) in having more ventrals (*t* = 6.74, *P* < 0.001), more subcaudals (*t* = -5.05, *P* = 0.002), more infralabials (9–11, mean 10.00 ± 0.89 in *Imantodes inornatus*), a longer head (head length/width 1.54–1.71, mean = 1.63 ± 0.07 in *Imantodes chocoensis* sp. n.; 1.29–1.61, mean = 1.45 ± 0.16 in *Imantodes inornatus*), and dark blotches on dorsum (dark spots and flecks in *Imantodes inornatus*; Fig. 3). The new species can also be distinguished from *Imantodes cenchoa* by having a single anal scale (vrs. two), fewer ventrals (*t* = 7.73, P < 0.001), fewer subcaudals (*t* = -4.04, P < 0.001), more infralabials (7–12, mean 9.92 ± 0.85 in *Imantodes cenchoa*), and dorsal dark blotches that include two or fewer vertebral scales and do not extend laterally onto ventrals (blotches are larger in *Imantodes cenchoa* and extend onto lateral tips of ventrals; Fig. 3). Scale counts and measurements of species of *Imantodes* from Ecuador are presented in Table 2.

**Table 2:** . Scale counts and measurements of species of *Imantodes* from Ecuador. Range (first line) and mean ± SD (second line) are presented when appropriate. Sample size is presented in parentheses if different from that in heading.

Character	*Imantodes cenchoa* N = 42	*Imantodes inornatus* N = 6	*Imantodes lentiferus* N = 30	*Imantodes chocoensis* sp. n. N = 7
Longitudinal scale rows on neck	17	17	15–17 15.07 ± 0.37	17
Longitudinal scale rows at midbody	17	17	15	17
Longitudinal scale rows anterior to cloaca	17	13–15 13.67 ± 1.03	15	17
Ventrals	249–280 262.62 ± 6.22	203–219 210.67 ± 5.99	216–237 226.80 ± 5.03	232–251 243.14 ± 5.84
Subcaudals	155–189 (37) 165.95 ± 8.01	109–126 (5) 117.80 ± 6.18	130–151 (27) 139.85 ± 5.89	140–161 (6) 151.83 ± 7.41
Anals	2	1–2 1.17 ± 0.41	1	1
Anterior temporals	1–3 2.13 ± 0.51	1–2 1.08 ± 0.29	1	1–2 1.43 ± 0.51
Posterior temporals	2–5 2.80 ± 0.53	1–2 1.92 ± 0.29	1–3 2.07 ± 0.36	2
Loreals	1	1	1	0
Preoculars	1–3 1.36 ± 0.53	1–2 1.25 ± 0.45	1–2 1.03 ± 0.18	1
Postoculars	1–4 2.11 ± 0.38	2–3 2.08 ± 0.29	1–2 1.81 ± 0.39	2–3 2.43 ± 0.51
Supralabials	7–9 7.99 ± 0.33	8	7–9 8.05 ± 0.34	9
Infralabials	7–12 9.92 ± 0.85	9–11 10.00 ± 0.60	9–12 10.68 ± 0.60	12–15 13.21 ± 0.80
Genials	2	2	2 (28)	2
Head length/width	1.35–1.80 1.56 ± 0.11	1.29–1.62 1.51 ± 0.13	1.37–1.91 1.62 ± 0.14	1.54–1.71 1.63 ± 0.07
Tail length/Total length	0.28–0.33 (37) 0.30 ± 0.01	0.27–0.30 (5) 0.28 ± 0.01	0.28–0.34 (27) 0.31 ± 0.01	0.29–0.32 (6) 0.31 ± 0.01
Maximum SVL (cm)	107.90	64.00	70.30	74.40
Maximum Total length (cm)	152.10	91.50	101.40	107.50

#### Description of holotype.

Male (Figs. 1, 2); SVL = 66.30 mm; tail length = 30.40 mm; head width = 7.98 mm; head length = 13.26 mm; head height = 5.37 mm.

Short, blunt head 1.7 times longer than broad and 2.5 times longer than deep; head abruptly distinct from neck, three times wider than thinnest part of neck and also slightly wider than greatest width of body; eye large and protuberant, occupying 27% of length of head, with elliptical pupil visible from anterior, lateral, dorsal, and ventral aspects; rostral 1.6 times wider than high, concave in anterior view, and narrowly visible from above; paired prefrontals extending anteroventrally to level of center of eye, each in contact with its mate and with frontal, supraocular, preocular, nasal, and internasal; frontal pentagonal, 1.6 times longer than wide (greatest width), and about 1.2 times longer than distance from its anterior edge to tip of snout; supraocular anteriorly narrow and posteriorly nearly as wide as greatest frontal width; broad parietals, about 1.3 times longer than wide; interparietal suture 1.2 times longer than length of frontal, and 1.4 times longer than distance from frontal to tip of snout; nasal plate single, centrally pierced by large naris (0.79 mm in diameter), in contact with rostral anteriorly, internasal dorsally, prefrontal posterodorsally, preocular posteriorly, and first and second supralabials ventrally; loreal absent; one large and high preocular; two postoculars (an extra tiny scale on left side ventrally), the lower somewhat less than half the size of the upper; temporals 2+2+3; supralabials 9, first and second in contact with nasal, fourth in contact with preocular, and fourth to seventh bordering the orbit; infralabials 13, with first six in contact with anterior genial, and sixth to eighth touching posterior genial; first pair of infralabials in contact medially behind mental; anterior and posterior genials nearly equal in length; gular scales with posterolateral apical pit.

Body higher than wide, rounded ventrolaterally; dorsal scales smooth, juxtaposed or subimbricate; dorsal body scales in 17 rows throughout; scales of vertebral row conspicuously enlarged, 2.5 times wider than adjacent dorsals, with concave posterior margins; ventrals 242; anal plate single; subcaudals 161.

**Color in preservative of holotype** (Figs 1, 2). Dorsal background light brown, with a longitudinal series of 63 dark brown middorsal blotches from head to cloaca; dark middorsal blotches longer anteriorly, 2–3 vertebral scales long, than posteriorly, 1–2 vertebral scales long, and extending laterally 1–3 (anteriorly) or more (posteriorly) dorsal scale rows, but never reaching ventral scales; each dark middorsal blotch irregularly bordered anteriorly and posteriorly by thin cream line; ventral aspect of body yellowish cream with dark brown spots and flecks; ventral aspect of tail yellowish cream with spots concentrating midventrally; dorsal surface of head light brown with several dark brown spots and two short dark stripes extending from posterior aspect of parietals to a point just posterior to head; ventral surface of head whitish cream.

**Figure 4.**
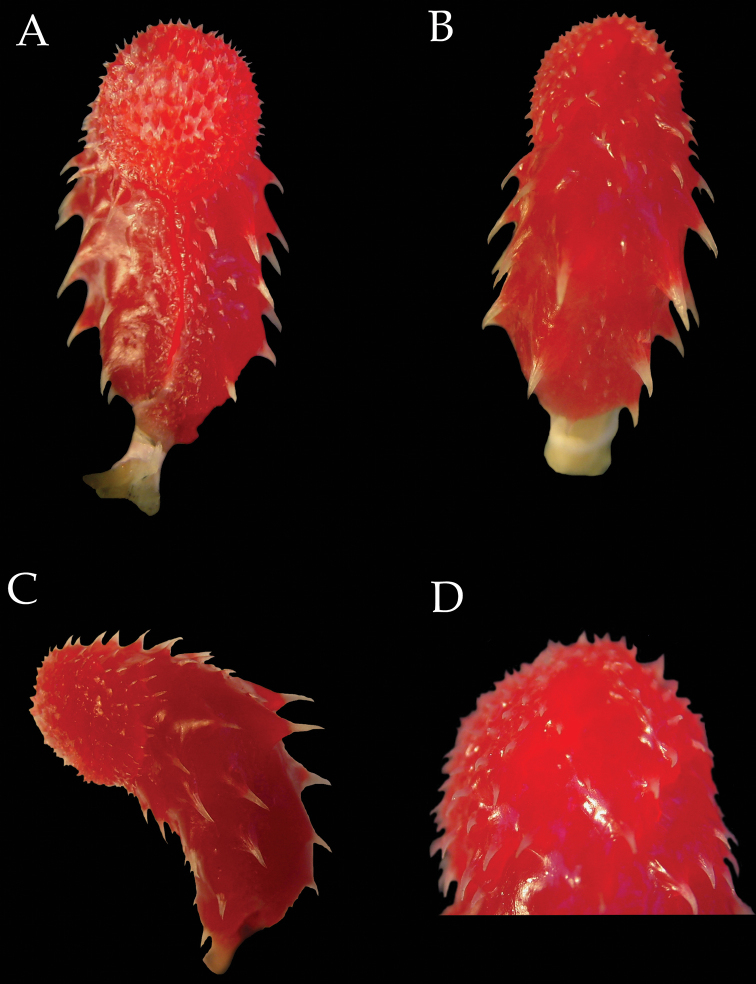
Right hemipenis of *Imantodes chocoensis* sp. n. (DHMECN 6753, paratype) in sulcal (**A**), asulcal (**B**), and lateral (**C**) views **D** close-up of distal end showing spines interrupted by sulci. Photographs by MYM.

**Hemipenes** (Fig. 4). The right hemipenis of the paratype DHMECN 6753 of *Imantodes chocoensis* was removed, fully everted and expanded (Fig. 4). The organ is bulbous and relatively long, 11.2 mm in length, and when adpressed to the outside of the tail it extends from the cloaca to the sixth subcaudal scale. The organ is longer than wide (width 46% of length), unilobed, symmetrical, calyculate, capitate, and arched towards the sulcal side. The sulcus spermaticus is simple, linear, semicentripetal, and thin, demarcated by thick bordering tissue at the base, particularly at the anterior border, and ending on the surface of the capitulum facing medially. The capitulum is ornamented with papillated calyces, spinulated proximally. The capitulum, approximately 45% the length of the hemipenis, is slightly demarcated by a groove, more prominent on the sulcal side and joining the sulcus spematicus. In the asulcate side the base of the capitulum has more prominent spines. Truncus covered by large spines, on the sulcate and asulcate and surfaces, 23 in total, 13 to the right of the sulcus spermaticus and 10 to the left, and has a few rows of small spines, two at the base on the asulcate side and two to three rows just right of the sulcus spermaticus.

#### Variation.

**Figure 5.**
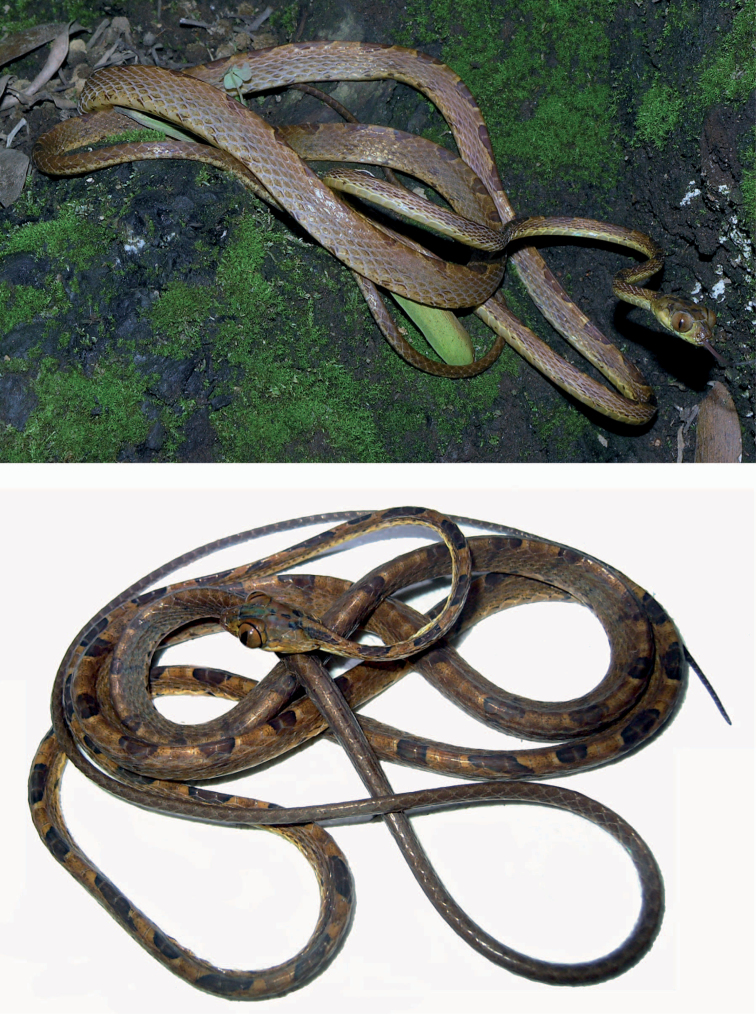
Paratypes of *Imantodes chocoensis* sp. n. UTA R-60205 (top), DHMECN 6753 (bottom). Photographs by ENS and MYM.

Intraspecific variation in scale counts and measurements in *Imantodes chocoensis* sp. n. is presented in Table 2. Color in life of paratypes UTA R-60205 and DHMECN 6753 (Fig. 5) is similar to color in preservative of holotype; iris copper brown. Middorsal blotches from head to cloaca vary between 55–66; one specimen (UTA R-60205) had one middorsal blotch covering five vertebral scales.

#### Distribution and ecology.

**Figure 6.**
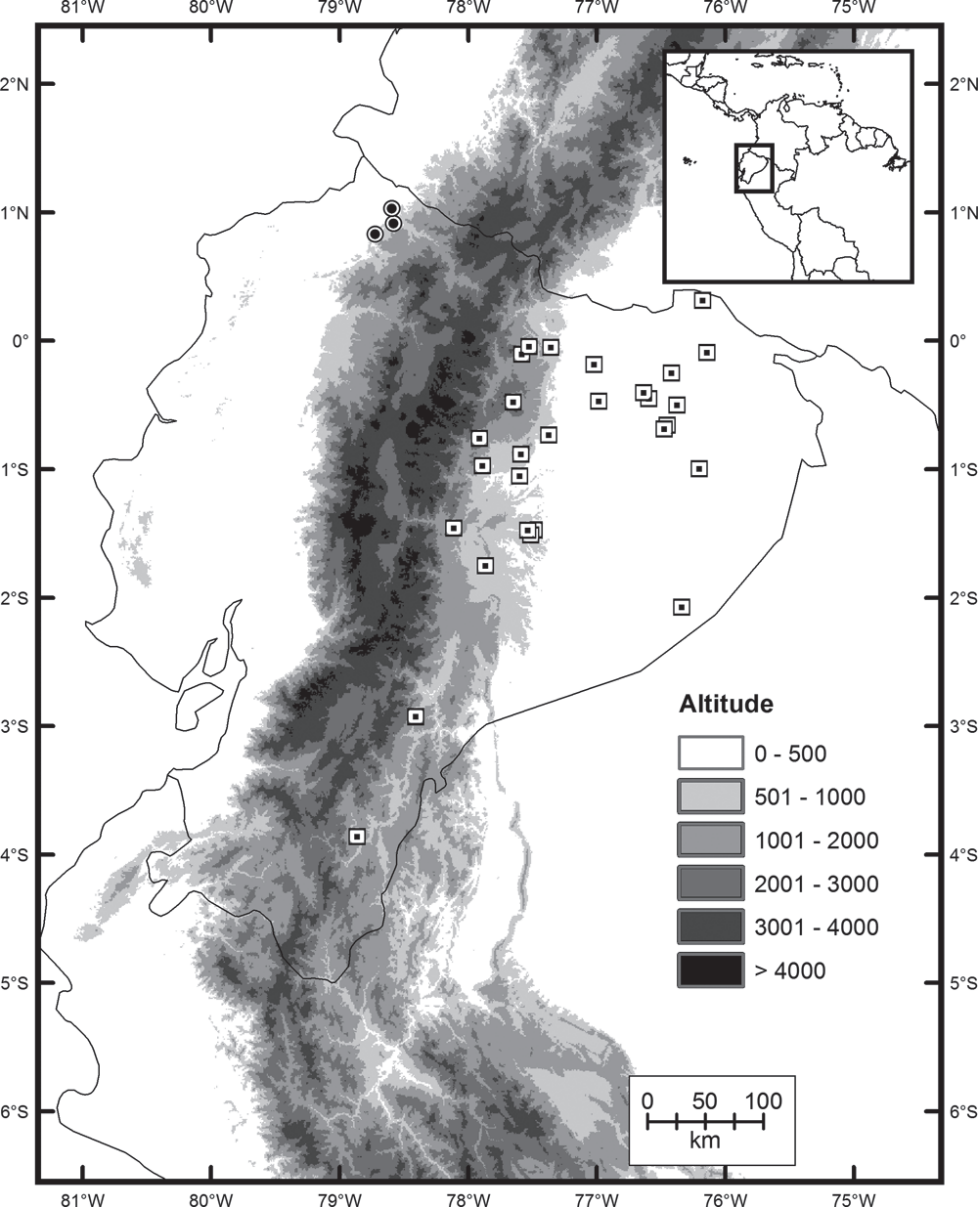
Distribution of *Imantodes chocoensis* sp. n. (circles) and its sister species *Imantodes lentiferus* (squares) in Ecuador.

*Imantodes chocoensis* inhabits Chocoan rainforests on the Pacific coast in northern Ecuador (Fig. 6). It occurs in lowland evergreen forest (Cerón et al. 1999) at elevations of 115–260 m in the provinces of Carchi and Esmeraldas. This new species has been collected in sympatry with *Imantodes cenchoa* in Esmeraldas, and most likely also shares its distribution with *Imantodes inornatus*. Other colubrid snakes collected in Tobar Donoso (Carchi) are *Chironius grandisquamis*, *Clelia clelia*, *Dendrophidion clarkii*, *Leptophis ahaetulla*, *Mastigodryas* sp., *Ninia atrata*, *Oxyrhopus petola*, *Pseustes shropshirei*, *Sibon nebulatus*, *Synophis bicolor*, *Tantilla melanocephala*, and *Xenodon rabdocephalus*. The known localities of *Imantodes chocoensis* lie in close proximity to the Ecuador-Colombia border and we expect for it to be found in neighboring Colombia.

#### Etymology.

The specific epithet *chocoensis* is an adjective derived from Chocó, the very humid tropical region comprising the Pacific coast of northern Ecuador, Colombia and Panama (Morrone 2001). This region is part of the 274,597 km^2^ Tumbes-Chocó-Magdalena hotspot as defined by Conservation International, which includes more than 320 species of reptiles.

#### Phylogenetic relationships.

**Figure 7.**
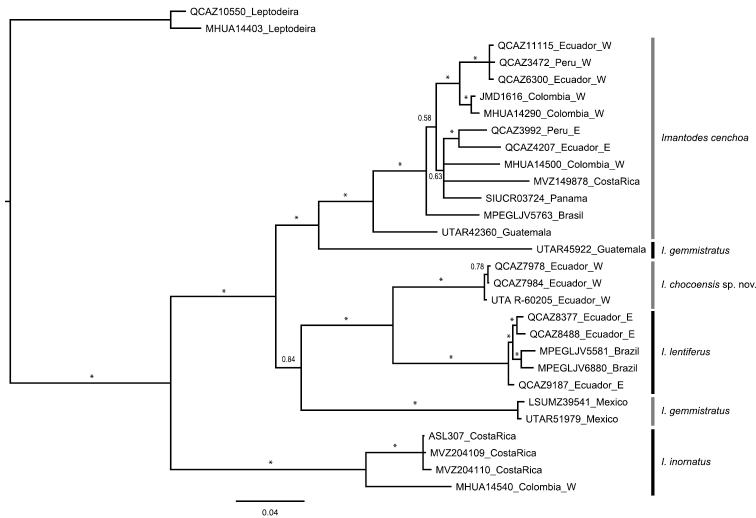
Majority rule (50%) consensus tree of 18,000 trees obtained from a Bayesian analysis of two mitochondrial genes (cyt-b, ND4) and 29 specimens. Asterisks correspond to posterior probability values > 0.99. Voucher numbers followed by country of collection are indicated for each terminal. E: east of the Andes, W: west of the Andes.

Selected models of evolution for sampled fragments of ND4 and cyt-b genes were HKY+I+G and TPM2uf+I+G, respectively. The resulting 50% majority rule consensus tree (Fig. 7) supports strongly (PP=1) a sister taxon relationship between *Imantodes chocoensis* sp. n. and *Imantodes lentiferus*, as well as the exclusivity (Rieppel 2010) of both species. Similarly, *Imantodes inornatus* and *Imantodes cenchoa* are recovered as exclusive clades with strong support (PP=1). Noteworthy the *Imantodes cenchoa* clade includes samples from Guatemala, Costa Rica, Panama, Brasil, and Colombia, Ecuador and Peru on both sides of the Andes. In agreement with previous hypotheses, *Imantodes gemmistratus* is paraphyletic; of three samples included in this study, one from Guatemala is sister to the *Imantodes cenchoa* clade with strong support (PP=1), whereas the other two, from Mexico, are weakly supported as sister to the *Imantodes chocoensis* sp. n. and *Imantodes lentiferus* clade.

## Discussion

Myers (1982) distinguished two monophyletic groups within *Imantodes* – *lentiferus* and *cenchoa* – based on hemipenial characters, maxillary dentition, relative tongue length, and coloration. According to Myers, the *lentiferus* group included *Imantodes lentiferus* and *Imantodes phantasma* as sister taxa, as well as *Imantodes inornatus*, whereas the *cenchoa* group included *Imantodes cenchoa*, *Imantodes gemmistratus* and *Imantodes tenuissimus*. Since the phylogenetic tree presented in this paper does not include all species of *Imantodes*, we cannot rigorously test Myers’ hypothesis of phylogenetic relationships within *Imantodes*. Nonetheless, two major differences are worth noting. First, in our phylogenetic tree *Imantodes inornatus* is sister to all other species of *Imantodes* (but see below). Second, in agreement with previous work (Daza et al. 2009), we recover a paraphyletic *Imantodes gemmistratus*, with specimens from Guatemala closely related to *Imantodes cenchoa* as postulated by Myers (1982), and specimens from Mexico in a clade with *Imantodes lentiferus* and the *Imantodes chocoensis* sp. n. (Fig. 7).

Monophyly of *Imantodes* remains controversial, but we refrained from testing it without better taxon and character sampling. Previous phylogenetic studies based on DNA sequence data have failed to support the monophyly of *Imantodes* as currently circumscribed (Daza et al. 2009; Mulcahy 2007). Except for a tree including only two species of *Imantodes* (Fig. 6 in Daza et al. 2009) and a Maximum Parsimony tree (Fig. 5 in Mulcahy 2007), these studies suggest that *Imantodes inornatus* is sister to a clade containing *Imantodes* and *Leptodeira* as sister taxa. Furthermore, the phylogenetic tree presented in this paper is congruent with this hypothesis (Fig. 7), suggesting that *Imantodes inornatus* might belong to a clade different from *Imantodes*. In fact, this species differs from other *Imantodes* in several morphological (e.g., no prominent dorsal blotches, or conspicuously enlarged vertebral scales; Fig. 3) and behavioral (e.g., head-flaring) features (Mulcahy 2007; Myers 1982).

### Key to the species of *Imantodes* from Ecuador

**Table:** 

1	Longitudinal scale rows at midbody 17	2
–	Longitudinal scale rows at midbody 15	*Imantodes lentiferus*
2	Vertebral scales 2.5–4 times wider than adjacent dorsal scales; dorsal color pattern with conspicuous dark blotches(Fig. 3)	3
–	Vertebral scales similar in size or slightly wider than adjacent dorsal scales; dorsal color pattern with dark spots and speckles (Fig. 3)	*Imantodes inornatus*
3	Loreal present; dorsal blotches include more than two vertebral scales and extend onto edge of ventrals (Fig. 3)	*Imantodes cenchoa*
–	Loreal absent; dorsal blotches include two or less vertebral scales and do not extend laterally onto ventrals (Fig. 3)	*Imantodes chocoensis*

## Supplementary Material

XML Treatment for Imantodes chocoensis

## Appendix

### Specimens examined

*Imantodes cenchoa* – COLOMBIA: *Meta*: UTA R-3364–65, Serranía de la Macarena, peak near Caño Sardinata, 30 Km WSW Vista Hermosa, 1300 ft; COSTA RICA: *Limón*: UTA R-12903, Approximately 17 km WSW Puerto Limón, between Río Blanco and Río Toro, 150 m; ECUADOR: *Carchi*: QCAZ 4419–20, Chical, road to San Pablo, 0.9211°N, 78.1809°W, 1248 m; *Cotopaxi*: QCAZ 1203, San Francisco de las Pampas, 0.4332°S, 78.9667°W, 1600 m; QCAZ 2771–72, 6 km west of Guasaganda, 0.8206°S, 79.1171°W, 350 m; QCAZ 8888, Naranjito, Bosque Integral Otonga (BIO), 0.4147°S, 79.0007°W; *Esmeraldas*: QCAZ 2248, La Mayronga, Lagarto, 1.0420°N, 79.2800°W; QCAZ 6669, Alto Tambo, El Placer, La Carolina, 0.7044°N, 78.2011°W, 500 m; QCAZ 7589–90, Bilsa, 0.6201°N, 79.9307°W; QCAZ 7930, Reserva Ecológica Bilsa, 0.3588°N, 79.7180°W, 590 m; UTA R-55946, road Alto Tambo - San Lorenzo, 1.33311°N, 78.61687°W, 336 m; UTA R-55947, road Lita - San Lorenzo, 1.07750°N, 78.65785°W, 91 m; UTA R-55948, Tunda Loma Lodge, 1.18333°N, 78.75349°W, 37 m; QCAZ 7979–81, 4 km N Durango 1.0283°N, 78.5950°W, 253 m; QCAZ 10203, near Lita, 0.8886°N, 78.5288°W; QCAZ 10706, lowlands of Reserva Ecológica Cotacachi Cayapas, Playa de Oro, Pote and Angostura estuaries, 0.8284°N, 78.7220°W; *Guayas*: QCAZ 9118, Bosque Protector Cerro Blanco, 2.1758°S, 80.0216°W, 213 m; *Morona-Santiago*: UTA R-37985–86, Limón; *Napo*: QCAZ 8422–23, río Hollín, 0.6950°S, 77.7307°W; *Orellana*: QCAZ 1742, 1752, Parque Nacional Yasuní, Block 16, Maxus road between Pompeya south and Iro, 0.6755°S, 76.3552°W, 110 m; QCAZ 3423, Parque Nacional Yasuní, km 80 road to Pompeya-Iro, Belle river bridge area, 0.8401°S, 76.3017°W; QCAZ 3650, Parque Nacional Yasuní, Estación Científica Yasuní, 0.6750°N, 76.3892°W, 220 m; QCAZ 8920–21, Florencia, 0.8966°S, 75.4370°W; QCAZ 9141, 9143, Parque Nacional Yasuní, road to Pompeya - Iro, km 10, 0.4598°S, 76.5931°W, 271 m; QCAZ 9528, southern bank of río Napo, Eden, 0.4983°S, 76.0711°W, 216 m; QCAZ 9551, northern bank of río Napo, San Vicente, 0.6790°S, 75.6511°W, 196 m; QCAZ 1738, Parque Nacional Yasuní, Block 16, Maxus road between Pompeya south and Iro, 0.6755°S, 76.3552°W, 100 m; *El Oro*: QCAZ 8992, Bella María, near Valle Hermoso, 3.5116°S, 79.8202°W, 282 m; *Pastaza*: QCAZ 4255, road to El Triunfo, Arajuno, 1.2325°S, 77.6876°W, 800 m; QCAZ 8161, around Villano, AGIP petroleum camp, K4, 1.4706°S, 77.4868°W; QCAZ 8378, around Villano, AGIP petroleum camp, K10 Unidad 3, 1.4727°S, 77.5359°W, 430 m; QCAZ 8914, Bataburo Lodge, 1.2500°S, 76.6667°W, 220 m; UTA R-15852, Río Cononaco, S side, within a few km of Perú border; *Pichincha*: EPN 1315354, Los Bancos, sector Milpe; *Santo Domingo de los Tsáchilas*: QCAZ 11048, La Concordia, Bosque Protector la Perla, 0.0570°S, 79.3590°W; QCAZ 11115, Hostería Tinalandia, near Alluriquín, 0.2965°S, 79.0523°W; *Sucumbíos*: QCAZ 1488–89, Reserva de Producción Faunística Cuyabeno, 0.2597°S, 75.8886°W; QCAZ 2564, Reserva de Producción Faunística Cuyabeno, research station (PUCE), 0.0018°S, 76.1755°W; GUATEMALA: *Alta Verapaz*: UTA R-46623, Finca Rubelpec, 650 m; UTA R-46624, Finca San Juan, 590 m; UTA R-46625, Finca San Juan, 550 m; UTA R-26194, N slope Sierra de lad Minas, Finca Pueblo Viejo, W slope Río Tinajas/Río Chiquito divide, 5.25 air km SSE, 1200–1500 m; UTA R-26193, N slope Sierra de las Minas, Finca Pueblo Viejo, 4.0 air km SE Pueblo Viejo, 0 m; UTA R-26195, Vicinity of Pueblo Viejo; *Baja Verapaz*: UTA R-42430, Purulhá, Finca Sabó, 15°14.87'N, 090°9.87'W, 1170 m; *Escuintla*: UTA R-28376, Finca Medio Monte, 720 m; UTA R-22804, 26198, S slope Volcán de Agua, Finca Rosario Vista Hermosa; *Huehuetenango*: UTA R-44705, 44716, Barillas, Finca Chiblac Buena Vista, 15°53.18'N, 091°14.73'W, ca. 930 m; UTA R-45491, Barillas, Finca Chiblac Buena Vista camino a Las Nubes, 15°52.12'N, 091°13.95'W, 1330 m; UTA R-42305, Sierra de Los Cuchumatanes, Finca Chiblac Buena Vista (now Aldéa Buenos Aires), 15°52.97'N, 091°14.80'W, 975 m; *Izabal*: UTA R-28373, E slope Montañas del Mico, 12.0 km WSW Puerto Santo Tomás, 786 m; UTA R-28375, E slope Montañas del Mico, along Río San Ramoncito, above Las Escobas, 226 m; UTA R-26205, El Estor, El Chupón, 2.0 m; UTA R-26206, El Estor, El Zapotillo, 2 m; UTA R-38217, Livingston, Aldéa La Libertad, Km 285 a Petén, 75 m; UTA R-39234, Livingston, Sierra de Santa Cruz, Cerro 1019, 940 m; UTA R-39536, Los Amates, Quirigua, Sitio Arqueológico; UTA R-29871, Los Amates, Sierra del Espiritu Santo, Aldéa San Antonio; UTA R-32996, Los Amates, Sierra del Espiritu Santo, Aldéa San Antonio, ca. 500 m; UTA R-28381, Los Amates, Sierra del Espiritu Santo, S side Cerro del Nylon, 725 m; UTA R-29870, Montañas del Mico, 11.6 km WSW Puerto Santo Tomás, 744 m; UTA R-16022–23, Montañas del Mico, 5.1 km WSW Puerto Santo Tomás, Las Escobas, 104 m; UTA R-20833–34, Montañas del Mico, 5.1 km WSW Puerto Santo Tomás, Las Escobas, 150–250 m; UTA R-28374, Montañas del Mico, 5.1 km WSW Puerto Santo Tomás, Las Escobas, 150 m; UTA R-29868–69, Montañas del Mico, 8 km WSW Puerto Santo Tomás, 457 m; UTA R-46686, Montañas del Mico, Crest of Cerro Las Escobas, near Guatel Tower, 860 m; UTA R-38216, 39535, Morales, Sierra de Caral, Aldéa Negro Norte, ca. 1150 m; UTA R-42358, Morales, Sierra de Caral, Aldéa Negro Norte, ca. 1100 m; UTA R-32998, Morales, Sierra de Caral, Aldéa Negro Norte, Cerro Negro Norte, 1180 m; UTA R-37240, Morales, Sierra de Caral, Carretera entre Quebradas y La Firmeza, 190 m; UTA R-42360, Morales, Sierra de Caral, Carretera Quebradas-La Firmeza, 990 m; UTA R-32997, Morales, Sierra de Caral, Cerro Bonillistas, 300 m; UTA R-38215, Morales, Sierra de Caral, La Firmeza, 825 m; UTA R-37241, Morales, Sierra de Caral, San Miguelito, 450 m; UTA R-37242, Morales, Sierra de Caral, San Miguelito, 470 m; UTA R-37243, Morales, Sierra de Caral, San Miguelito, 600 m; UTA R-37244, Morales, Sierra de Caral, San Miguelito, 550 m; UTA R-37245, Morales, Sierra de Caral, San Miguelito, 560 m; UTA R-46687, Morales: Sierra de Caral: Finca “San Silvestre”, 475m; UTA R-37239, Municipio de Morales, Sierra de Caral, along tributary of Río Bobos, E of San Miguelito, 625 m; UTA R-37238, Municipio de Morales, Sierra de Caral, road between Quebradas and San Miguelito, 100 m; UTA R-20832, Near Mariscos, 20 m; UTA R-46685, Polochic Valley, 4 mi w El Estor, 30 m; UTA R-42361, Quebrada El Branchi, 2.0–4.0 km NE Aldéa La Libertad, 50 m; UTA R-22805, Seshán, 900 m; UTA R-26207, Sierra de Santa Cruz, Cerro 1019, 980; UTA R-46681, Sierra de Santa Cruz, Exmibal Forest (first crest on road from El Estor to Finca Semuc) just W of El Estor, 560 m; UTA R-46682, Sierra de Santa Cruz, Exmibal Forest (first crest on road from El Estor to Finca Semuc) just W of El Estor, 650 m; UTA R-46683, Sierra de Santa Cruz, Exmibal Forest (first crest on road from El Estor to Finca Semuc) just W of El Estor, 650 m; UTA R-46684, Sierra de Santa Cruz, Exmibal Forest (first crest on road from El Estor to Finca Semuc) just W of El Estor, 805 m; UTA R-28377, Sierra de Santa Cruz, Finca Chacchilá, El Coco, ca. 300 m; UTA R-22168, 32999, Sierra de Santa Cruz, Finca Semuc; UTA R-28379, Sierra de Santa Cruz, Finca Semuc headquarters, ca. 500 m; UTA R-26199, Sierra de Santa Cruz, Finca Semuc, 1.0 km S headquarters, 445 m; UTA R-28378, Sierra de Santa Cruz, Finca Semuc, 1.0 km S headquarters; UTA R-29875, Sierra de Santa Cruz, Finca Semuc, 2 km S headquaters, Río Semuc, 400 m; UTA R-42359, Sierra de Santa Cruz, Finca Semuc, Casco, ca 450 m; UTA R-26196, Sierra de Santa Cruz, Finca Semuc, Chinamococh, 650 m; UTA R-26197, Sierra de Santa Cruz, Finca Semuc, Chinamococh, 600 m; UTA R-26200, Sierra de Santa Cruz, Finca Semuc, Chinamococh, 675 m; UTA R-29874, Sierra de Santa Cruz, Finca Semuc, S side Cerro Serujijá, 700 m; UTA R-29872, Sierra de Santa Cruz, Finca Semuc, S side Semococh, 580 m; UTA R-29873, Sierra de Santa Cruz, Finca Semuc, S side Semococh, 860 m; UTA R-26201, Sierra de Santa Cruz, S side Cerro Cana Tomasa, 475 m; UTA R-26202–04, Sierra de Santa Cruz, S side Cerro Cana Tomasa, 400 m; UTA R-26208–10, Sierra de Santa Cruz, Xiacam, 980 m; *Petén*: UTA R-39231, 7.9 km SW (by road) El Cruce, 740 m; UTA R-39233, ca. San José, N shore Lago Petén-Itzá; UTA R-50300, Gringo Perdido, NE side of Lago Petén-Itzá, near El Remate, 140 m; UTA R-46124, La Libertad, Parque Nacional, Sierra Lacandón, Distrito Yaxchilán; UTA R-39232, Near El Caoba, 400 m; UTA R-50299, on trail starting 1.6 km S of Tikal Visitor’ S Center; *Quezaltenango*: UTA R-20835, 22802–03, S slope Volcán Santa María, Finca El Faro, ca 4 km N El Palmar, 875 m; *San Marcos*: UTA R-42285, Area La Trinidad-Aldéa La Fraternidad, 1200–1940 m; UTA R-45685, Malacatán, Finca San Ignacio, 14°56.75'N, 092°2.50'W, 610–760 m; UTA R-39510, Municipio Esquipulas Palo Gordo, Aldéa Fraternidad, Finca La Esperanza, 1550–1890 m; UTA R-39329–35, San Rafael Pie de la Cuesta, Finca America El Vergel, ca. 1500 m; UTA R-45683–84, San Rafael Pie de la Cuesta, Finca America El Vergel, 14°56.30'N, 091°53.48'W, 1600 m; *Sololá*: UTA R-45687, San Lucas Tolimán, Finca Santo Tomás Pachuj, 14°36.53'N, 091°7.40'W, 1340 m; *Zacapa*: UTA R-32994, Sierra del Merendón, Finca San Enrique, Sur del Casco; UTA R-32995, Sierra del Merendón, Finca San Enrique, Sur del Casco, 800 m; UTA R-32993, Sierra del Merendón, Finca San Enrique, Sur del Casco, Cerro La Palmichera, 890 m; *Unknown*: UTA R-22167, No specific locality; HONDURAS: *Olancho*: UTA R-53268, Sierra de Agalta, 14.945N, 86.151W, 1195 m; *Unknown*: UTA R-24863, 25040–45, 24860–62, No specific locality; MEXICO: Nayarit: UTA R-53413, 12.4 road miles N of Las Varas, 21.21270°N,104.99162°W, 749 m; *Oaxaca*: UTA R-2461, 13.5 from Veracruz-Oaxaca state line, 52 mi N La Ventosa, 17°10.80'N, 095°3.60'W; UTA R-52643, Municipio La Soledad, Piedra Ancha, 16.75417N, 95.45699W, 1135 m; UTA R-25819, Sierra Juárez, between Metates and Vista Hermosa, 17°40.20'N, 096°19.20'W, 900–1500 m (1200 m); UTA R-12336, Sierra Juárez, Metates, 17°42.00'N, 096°18.60'W, 902 m; UTA R-14148, Sierra Juárez, Metates, 17°42.00'N, 096°18.60'W; UTA R-12337, Sierra Juárez, Metates, 17.7 km S Valle Nacional, 17°42.00'N, 096°18.60'W, 914 m; *Quintana Roo*: UTA R-53411, Mex 180D, 21.05151°N, 87.06756°W, 13 m; *San Luis Potosí*: UTA R-4673, Hwy 85, 8.0 mi S Antiqua Morelos; *Tamaulipas*: UTA R-4672, Hwy 80, 1.0 mi E Nuevo Morelos; *Veracruz*: UTA R-3013, 0.2 mi N Los Mangos, 18°16.20'N, 095°7.20'W, 400 m; UTA R-3011, 0.2 mi N of Encinal, 18°15.00'N, 095°6.60'W, 370 m; UTA R-9410, 1.8 mi S Juan Diaz Covarrubias, 18°7.80'N, 095°9.60'W; UTA R-3017, 10 mi S Catemaco (bridge), 18°17.40'N, 095°7.20'W, 450 m; UTA R-3015, 2.4 mi S Los Mangos, 270 m; UTA R-3012, 3.2 mi S Catemaco (bridge), 18°22.20'N, 095°7.20'W, 400 m; UTA R-3016, 7.8 mi N Juan Diaz Covarrubias, 18°12.60'N, 095°7.20'W.

*Imantodes gemmistratus* – COSTA RICA: *Guanacaste*: UTA R-44972, Liberia, Santa Rosa National Park; GUATEMALA: *Escuintla*: UTA R-22807, Finca Medio Monte, ca. 500 m; UTA R-22169–70, 22806, S slope Volcán de Agua, Finca Rosario Vista Hermosa; *San Marcos*: UTA R-45922, Malacatán, Finca San Ignacio, 14°56.75'N, 092°2.50'W, 610–760 m; UTA R-28380, San Pablo, Finca Palmira; *Santa Rosa*: UTA R-45651, Taxisco, Aldéa Madre Vieja (entre Monterrico and Ixtapa), 0 m; *Sololá*: UTA R-4496, San Juan Obispo; *Unknown*: UTA R-4706, No specific locality; MEXICO: *Guerrero*: UTA R-53968, Carretera Marquelia - San Luis Acatlán, 16.68388°N, 98.78722°W; *Jalisco*: UTA R-53967, Carretera Puerto Vallarta - La Cumbre Mex 200, 19.4 road miles S of Puerto Vallarta Plaza; *Nayarit*: UTA R-53539, Carretera Puerto Vallarta - Tepic, Mex 200, 21.20688°N, 104.95229°W, 86m; UTA R-53540, MX Hwy 200 S of Tepic, just S of Las Varas, 21.08391°N, 105.20233°W, 24 m; UTA R-53966, Carretera Tepic Las Vacas, Mex 200. 17.3 road miles N of Las Vacas, 21.21319°N, 104.94702°W, 879 m; *Oaxaca*: UTA R-52646, Camino Niltepec-El Palmar, 16.6808N, 94.57074W, 230 m; UTA R-6638, Cerro Baúl, 16°33.60'N, 094°10.20'W, 1700 m; UTA R-2460, 23.3 mi E La Ventosa, 16°33.60'N, 094°36.60'W; UTA R-8828, 5.9 mi S El Tejocotes, 17°13.20'N, 097°3.00'W; UTA R-6072, 8.0 km NE Tapanatepec, 16°24.60'N, 094°9.60'W, 274 m; *Sinaloa*: UTA R-4823, 3.6 mi E Concordia; UTA R-4376, 5.0 mi SE Villa Unión, 100 ft; *Veracruz*: UTA R-3014, 5.5 mi S Catemaco (bridge), 18°21.00'N, 095°6.60'W, 540 m; UTA R-3074, Los Tuxtlas, 3.6 mi S Catemaco, 18°22.80'N, 095°7.20'W, 390 m; UTA R-3081, On way back from Encinal; UTA R-6869, 2.1 mi N Juan Diaz Covarrubias, 18°9.60'N, 095°9.60'W; UTA R-6865, 4.4 mi N Juan Diaz Covarrubias, 18°10.20'N, 095°8.40'W; UTA R-9411, 5.4 mi S Juan Diaz Covarrubias, 18°10.80'N, 095°7.80'W; *Yucatan*: UTA R-53969, Mex 180D, 20.74248°N, 88.21232°W.

*Imantodes inornatus* – COSTA RICA: *Cartago*: UTA R-12904, 2.0 km W Pavones de Turrialba, 500 m; ECUADOR: *Esmeraldas*: QCAZ 3843, La Chiquita protected forest, 30 km San Lorenzo-Ibarra, 1.2333°N, 78.7600°W; DHMECN 5661, road San Lorenzo-Lita, 1.1979°N, 78.7305°W, 60 m; UTA R-55930–31, road San Lorenzo-Lita, 1.26219°N, 78.79528°W, 40 m; *Manabí*: EPN 8978, Maicito on road to Chone.

*Imantodes lentiferus* – COLOMBIA: *Vaupés*: UTA R-5175, Timbó; ECUADOR: *Morona Santiago*: QCAZ 9187, General Leonidas Plaza Gutiérrez (Limón), Napinaza, Quebrada río Napinaza, 2.9230°S, 78.4080°W; *Napo*: QCAZ 284–85, El Reventador, 0.0412°S, 77.5268°W; DHMECN 4591, Archidona, Cotundo, Narupa Biological Reserve, Fundación Jocotoco, 0.7583°S, 77.9103°W, 1800 m; *Orellana*: QCAZ 8476, Pata 3 and Chontayacu, Huataracu community, 0.1811°S, 77.0201°W, 346 m; QCAZ 10110, Parque Nacional Yasuní, 1 km E road to Maxus, km 38, 0.6539°S, 76.4518°W; *Pastaza*: QCAZ 8170, around Villano, AGIP petroleum camp, K4 Unidad 1, 1.4722°S, 77.4864°W; QCAZ 8197, around Villano, AGIP petroleum camp, K4 Unidad 3, 1.4697°S, 77.4874°W; QCAZ 8276, around Villano, Kurintza community, Kurintza Unidad 3, 1.5100°S, 77.5141°W, 387 m; QCAZ 8377, around Villano, AGIP petroleum camp, K10 Unidad 3, 1.4727°S, 77.5359°W, 430 m; DHMECN 4368, Montalvo, Nuevo Corrientes, Kurintza, 2.0721°S, 76.3397°W, 300 m; EPN 1276, Mera, 1.4567°S, 78.1114°W; EPN 6256, Arajuno, Parroquia Curaray, caserío Chuyayacu, 0.4744°S, 77.6505°W; EPN 6482, Arutam, 1.7500°S, 77.8666°W; EPN 8974, Shell, Taigsha; EPN 897677, no specific locality; *Sucumbíos*: EPN 11531, 11617, Gonzalo Pizarro, Lumbaqui, Aguarico protected Forest, 0.0492°S, 77.3567°W; DHMECN 160, Lago Agrio, Tarapoa, San Pablo de Kantesiya, 0.2499°S, 76.4166°W, 300 m; DHMECN 1572, Shushufindi, Limoncocha, 0.3999°S, 76.6333°W, 300 m; DHMECN 8345, Putumayo, Santa Elena, Block 27, 0.3667°N, 76.1869°W, 264 m; *Zamora Chinchipe*: QCAZ 8488, Guadalupe, Afluente del río Piuntza, Finca de Mesías San Martín, 3.8564°S, 78.8646°W, 1154 m; [*Pichincha*]: EPN 8975, Pachijal (in error), 0.1300°S, 78.7264°W; NO SPECIFIC POLITICAL UNIT: *Oriente*: EPN 897273, *No locality data*: EPN 9585.

*Imantodes tenuissimus* – MEXICO: *Quintana Roo*: UTA R-53970, Mex Hwy180D, 21.07407°N, 87.02190°W, 17 m; *Yucatan*: UTA R-53412, Carretera Yaxacalba - Tahdzibichen, 20.52230°N, 88.82845°W, 33 m.
